# Unveiling Molecular Mechanisms Underlying Obesity‐Associated Systemic Lupus Erythematosus

**DOI:** 10.1002/edm2.70077

**Published:** 2025-09-21

**Authors:** Roshvin Kailashnath Pillai, Rishvini Kailashnath Pillai, Vinibha Rajakumari Illankovan, Vinod Balasubramaniam, A. M. Alabsi, Anupam Biswas, Hari Kumar Darnal, Saminathan Kayarohanam, Madhan Kumar Soutallu Janakiram, Vetriselvan Subramaniyan

**Affiliations:** ^1^ Faculty of Medicine MAHSA University Jenjarom Selangor Malaysia; ^2^ Centre for Pre‐University Studies MAHSA University Jenjarom Selangor Malaysia; ^3^ Jeffrey Cheah School of Medicine and Health Sciences Monash University Malaysia, Jalan Lagoon Selatan, Bandar Sunway Subang Jaya Selangor Malaysia; ^4^ Management and Science University University Drive, Off Persiaran Olahraga Shah Alam Selangor Malaysia; ^5^ Faculty of Medicine, Department of Physiology AIMST University Bedong Kedah Malaysia; ^6^ International Medical School Management and Science University Shah Alam Selangor Malaysia; ^7^ Faculty of Bioeconomics, Food & Health Science University Geomatika Malaysia, Prima Peninsula Kuala Lumpur Malaysia; ^8^ Department of Anatomy, Faculty of Medicine Manipal University College Bukit Baru Malaysia; ^9^ Department of Biomedical Sciences Sir Jeffrey Cheah Sunway Medical School Faculty of Medical and Life Sciences Sunway University Subang Jaya Selangor Malaysia

**Keywords:** autoimmune disease, immune dysregulation, metabolic reprogramming, obesity‐induced inflammation, systemic lupus erythematosus (SLE)

## Abstract

**Background:**

Systemic lupus erythematosus (SLE) is a chronic autoimmune disease primarily affecting women of childbearing age, characterised by relapsing inflammation across multiple organ systems. Its aetiology involves genetic and environmental factors that trigger immune dysregulation, leading to the excessive release of autoantibodies.

**Objective:**

This study discusses the pathogenesis, diagnosis, management and emerging therapeutic targets in SLE, with a focus on metabolic reprogramming and the role of obesity.

**Methods:**

Diagnosis is based on clinical and laboratory findings, with the EULAR and ACR criteria being the most advanced. SLE management requires individualised treatment, addressing the severity and organs affected.

**Results:**

SLE presents with symptoms ranging from mild skin rashes to severe conditions like pulmonary hypertension and kidney failure. Advances in care have improved outcomes, with 80%–90% of patients achieving normal life expectancy with proper treatment. Metabolic reprogramming in immune cells is crucial to SLE pathogenesis, as altered glycolysis and fatty acid oxidation contribute to inflammation.

**Discussion:**

Obesity is recognised to aggravate SLE by fostering chronic inflammation, immunological dysregulation and dysbiosis of the gut microbiota. These situations may exacerbate autoimmunity, especially in genetically predisposed individuals. It is suggested that obesity significantly contributes to the pathogenesis of SLE, and additional study should investigate metabolic therapy aimed at obesity and microbiota to re‐establish immunological equilibrium and mitigate disease development.

**Conclusion:**

Targeting metabolic pathways may offer new therapeutic options for improved disease management. Particular abnormalities in gut microbiota, characterised by reduced diversity and an increase in pro‐inflammatory species, influence obesity‐related immunological dysregulation in systemic lupus erythematosus and may present new therapeutic targets.

## Graphical abstract

Obesity exacerbates immunological dysfunction in systemic lupus erythematosus through metabolic reprogramming of immune cells. Modified glycolysis and fatty acid oxidation promote chronic inflammation. Decreased microbial diversity and heightened pro‐inflammatory species exacerbate disease progression. Targeting metabolic and microbial pathways may enhance systemic lupus erythematosus outcomes in obese people.

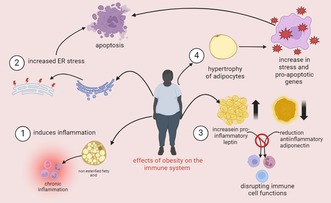

## 1 Introduction

Systemic lupus erythematosus (SLE) is a systemic autoimmune disorder that requires lifelong treatment with drug classes such as glucocorticoids, hydroxychloroquine and immunosuppressive agents [1]. It is a chronic autoimmune disease affecting multiple organ systems, characterised by a relapsing and remitting course, with a significantly higher prevalence in women of childbearing age, exhibiting a female‐to‐male ratio of 9:1 [2]. The typical clinical presentation of SLE is young women and includes red rashes on the skin and multi‐organ involvement. The aetiology of SLE may be related to genetic predispositions, exposure to the environment or triggers that are endogenous in nature [3]. Systemic lupus erythematosus (SLE) is an autoimmune disorder marked by the generation of antibodies that attack nuclear and cytoplasmic antigens. Environmental and genetic factors are recognised to combine and provoke aberrant immunological responses, resulting in increased pathogenic autoantibody synthesis by B cells, cytokine dysregulation and consequent tissue and organ damage [4]. There is a broad range of clinical features seen in SLE. Ranging from mild cutaneous involvement to a more severe presentation such as pulmonary hypertension, cardiac failure and kidney failure [5]. The diagnosis of systemic lupus erythematosus (SLE) is based on clinical and laboratory findings. The latest classification criteria developed by the European League Against Rheumatism (EULAR) and the American College of Rheumatology (ACR) are considered the most advanced and accurate criteria to date [5].

Managing systemic lupus erythematosus is difficult due to its multisystem involvement and unpredictable progression. Significant organ impairment, particularly affecting the kidneys, lungs or heart, can diminish life expectancy. Obesity may exacerbate systemic lupus erythematosus by fostering chronic inflammation, immunological dysregulation and gut dysbiosis, resulting in increased flare‐ups and diminished remission periods. It is proposed that obesity significantly influences the severity of SLE, and research should concentrate on metabolic therapies to reestablish immunological equilibrium and enhance outcomes. However, with diligent monitoring and care, approximately 80%–90% of SLE patients can achieve a normal life expectancy [5]. In order to maintain the correct operation of the cell, cellular metabolism is a complicated and dynamic process that involves taking up available nutrients in the cellular environment and generating new metabolites. The metabolic needs of a cell vary based on its activity phase. Utilising mitochondrial processes including fatty acid oxidation and the tricarboxylic acid cycle (TCA cycle), quiescent cells produce energy. These routes enable continuous energy synthesis in long‐lived cells and are very effective in producing adenosine triphosphate (ATP). Metabolic reprogramming takes place to adapt to the needs of the cell upon activation. Immune responses have developed to be energetically expensive, even impacting homeothermy and other maintenance processes, leading to trade‐offs in physiology [6]. Using glycolytic pathways, activated cells quickly generate large quantities of ATP to power cell division and proliferation. Compared to mitochondrial catabolic processes, glycolysis is less effective in producing energy, producing two moles of ATP for every unit of glucose [6]. Nevertheless, because glycolysis does not require mitochondrial development, it may be increased more quickly than mitochondrial metabolic pathways, offsetting this inefficiency. Furthermore, glycolysis is a predominant metabolic route in active cells because it produces biosynthetic intermediates that may be used for ribose synthesis and other vital processes [7]. In a stable state, the metabolic profile of native immune cells is quiescent. The activation of these cells is triggered by antigen contact or inflammatory cytokine signals, resulting in metabolic reprogramming [6, 7]. Fatty acids are initially converted to fatty acid acyl‐CoA in the cytosol, and they are subsequently broken down by β‐oxidation in the mitochondria to produce acyl‐CoA, FADH2 and NADH, which may be utilised to produce ATP. Promotion of antimicrobial macrophage function is seen when there is inhibition of fatty acid oxidation [1].

### 1.1 Obesity‐Driven Dysregulation of Immune Homeostasis

Individuals who are above 30 kg/m^2^ in the body mass index scale are classified as obese. In developed countries, it has become a major health issue. These individuals develop conditions that increase their risk of non‐communicable diseases such as type 2 diabetes, hypertension, cardiovascular diseases, cancer and asthma. The most important factor in this dysregulation is the fact that more energy is being taken up than being burnt. This leads to decreased blood flow and oxygen availability in the adipose tissues, cell death and mechanical stress on connective tissues as the fat mass increases. A low‐grade chronic inflammatory response arises in conjunction with the gut's increased permeability and the spread of bacterial products, which causes peripheral and localised dysregulation of T cell polarisation [6]. Obesity causes significant alterations in adipose tissue, such as adipocyte hypertrophy, extracellular matrix enlargement and wide‐ranging modifications in the immune cell compartment. Immune‐mediated alterations during obesity also affect the function of other tissues, including the brain, liver, muscles and pancreas. An intricate network of immune cells makes up the heart and vasculature. They are responsible for preserving homeostasis, responding to ischaemic, infectious and non‐infectious injury and occasionally unintentionally spreading autoimmune diseases or other pro‐inflammatory insults, such as obesity‐related chronic inflammation [7]. A recent study demonstrated the increased severity and worse prognosis following infection with severe acute respiratory syndrome coronavirus 2 (SARS‐CoV‐2) in obese patients compared to their lean counterparts, highlighting the influence of the inflammatory state that obesity generates throughout the body [8].

## 2 Molecular Pathogenesis of Adipose Tissue Inflammation

Autoimmunity caused by excess adipose tissue results in altered T‐cell subsets, dysregulated adipokines and elevated autoantibody synthesis. The Toll‐like receptor (TLR) family is pivotal in connecting inflammation with obesity and insulin resistance in humans and rats [9]. Out of the 10 human TLR members, it has been revealed that TLR2, TLR4, TLR5 and TLR9 regulate insulin resistance and metabolic inflammation in the liver and adipose tissue. Transmembrane receptors that have undergone evolutionary conservation, or TLRs, are essential for both innate and adaptive immunity. They do this by identifying different microbial components and initiating signalling pathways that are crucial for inducing inflammatory responses. TLRs sense external microbial ligands as well as endogenous chemicals secreted from dead or injured cells. They also govern a number of sterile inflammatory processes. Mammalian endosomal TLRs, specifically TLR7, TLR8, TLR9 and TLR3, are crucial in the development of systemic lupus erythematosus (SLE) in humans [10].

In humans, single‐stranded RNA (ssRNA) is sensed by both TLR7 and TLR8, which have a common evolutionary background. However, in mice, ssRNA is exclusively sensed by TLR7. Even though it does not appear to have a ligand, research has shown that TLR8 in mice regulates TLR7‐mediated lupus, which indicates that it is physiologically significant. Because TLR7 expression and DC signalling are enhanced in TLR8‐deficient animals on the C57BL/6 background, lupus develops [10]. To prevent inflammatory pathologies and SLE, TLR7 must be strictly regulated and controlled. There has never been any information published on the role that TLR7 plays in the long‐term inflammation of SLE in relation to obesity or metabolic syndrome [10, 11]. TLR7 may be the connection between metabolic syndrome and SLE because of the elevated risk of metabolic syndrome in SLE patients and its association with the onset of SLE. Data from Hanna Kazazian N et al. showed that HFD enhanced the incidence of metabolic problems and accelerated the onset of SLE in TLR8ko mice [11]. TLR7 expression and signalling in DCs were upregulated in conjunction with the prolonged illness course observed in HFD‐fed TLR8ko animals. TLR7/8ko animals were completely shielded from metabolic syndrome and SLE, in contrast to TLR8ko mice. The link between SLE and metabolic illness is thus associated with TLR7 signalling [11]. Metabolic reprogramming in immune cells, characterised by increased glycolysis and diminished oxidative phosphorylation, induces hyperactivation of T cells and dendritic cells via critical signalling pathways, including mTOR, AMPK and HIF‐1α. The dysregulation of these pathways enhances the secretion of inflammatory cytokines and the formation of autoantibodies, connecting metabolic stress to immunological dysfunction in obesity‐related systemic lupus erythematosus.

### 2.1 Adipokines and Cytokine Crosstalk in Lupus Development

The heterogeneous class of chemicals known as adipokines, which includes the cytokine resistin, adiponectin and leptin, is strongly linked to metabolic syndrome. Adiponectin has anti‐inflammatory and anti‐fibrotic effects. One characteristic that SLE patients frequently exhibit is the dysregulation of cytokines and adipokines [12]. Hyperphagia, weight gain and insulin resistance are shown in mice with loss of function of the ob gene; these symptoms resolve when exogenous leptin is given. The 16‐kDa nonglycosylated protein leptin is produced in large quantities by subcutaneous White Adipose Tissue (WAT). Leptin's structure is akin to that of granulocyte‐colony stimulating factor (G‐CSF) and IL‐6, with a bundle of four α‐helices maintained by cysteine disulfide bonds. The class I cytokine receptor family's sole membrane‐spanning leptin receptor (LEPR in humans, Ob‐R in mice) is the receptor via which leptin transmits its signals [13, 14]. The leptin receptor has six known isoforms: the long form (ObRb), four isoforms with short cytoplasmic tails (ObRa, ObRc, ObRd and ObRe), and one soluble form (ObRf). Three conserved tyrosine residues found in the entire cytoplasmic tail of ObRb can affect downstream signalling [14].

### 2.2 Immune Dysregulation With Leptin

Leptin regulates energy balance and influences other elements of immunological function in addition to this. Leptin levels fall during fasting or starvation, which impairs cell‐mediated immunity, including T cell mitogen responses and delayed‐type hypersensitivity reactions. Immune cells express both the long (ObRb) and short (ObRa) forms of the leptin receptor, according to research by Gainsford et al. Additionally, the same study demonstrated that clonal immune cell line growth was promoted by leptin binding to the ObRb [15]. Furthermore, research has been done on how leptin affects innate and adaptive immune system cells. ObRa and ObRb are expressed by monocytes and macrophages. Leptin stimulates the activation markers and inflammatory cytokines (TNF‐α and IL‐6) and promotes the proliferation of monocytes. Because neutrophils only carry the short form of the receptor (ObRa), they are not able to perform any downstream signalling [16].

**FIGURE 1:**
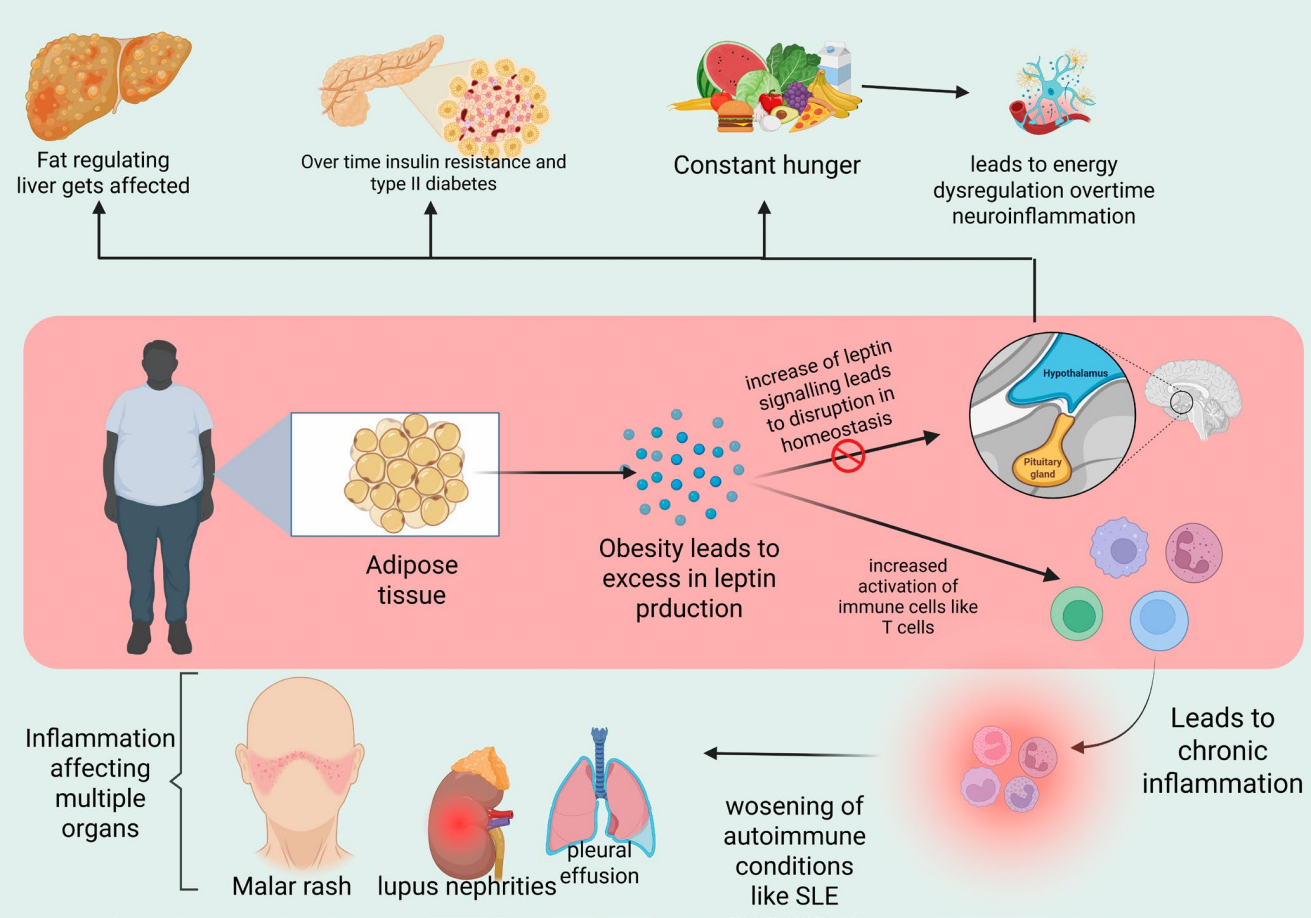
Obesity‐induced leptin excess disrupts homeostasis, triggers immune activation, and drives inflammation and metabolic dysfunction.

Leptin‐incubated neutrophils exhibit delayed apoptosis; the anti‐apoptotic effect was attributed to MAPK signalling downstream of ObRa. The fact that Kamp et al. found that supraphysiological doses of leptin were required to produce these effects in neutrophils raises questions about the biological significance of this and other investigations. Moreover, leptin stimulates neutrophil chemotaxis. When leptin is administered intraperitoneally to mice, neutrophils migrate to the peritoneum; however, the mechanism is probably an indirect effect of leptin causing monocytes and macrophages to produce TNF‐α and chemokines [17]. Leptin inhibits the surface expression of L‐selectin and ICAM‐3 in eosinophils, while it increases the expression of the adhesion molecules ICAM‐1 and CD18. Additionally, leptin causes eosinophils to produce inflammatory cytokines such as IL‐1β, IL‐6 and MCP‐1 [17]. DCs that are immature or mature express ObRb. ObRb is surface upregulated in response to exogenous leptin treatment in vitro, although surface activation marker expression remains unaltered. On the other hand, DCs treated with leptin produce more chemokines and cytokines. Furthermore, leptin‐treated DCs have been shown to more effectively activate heterologous T cells and encourage naïve T cells to undergo Th1 cell differentiation [18]. The leptin receptor is expressed by both naïve and activated T cells; however, T cell expression of the leptin receptor is further upregulated during stimulation or activation (Figure 1).

### 2.3 Autoimmunity in Leptin

Rheumatoid arthritis (RA), multiple sclerosis (MS), autoimmune disease models and type 1 diabetes are all prevented in mice lacking leptin signalling. The pro‐inflammatory action of leptin is reflected in the dysregulation of the CD4^+^ T cell subset in many autoimmune disorders, which includes increased Th1 and Th17 responses and reduced Treg responses [19]. Compared to C57BL/6 mice, ob/ob mice that have SLE produced by the hydrocarbon oil pristane have increased Treg and no autoantibodies. The NZBWF1 mouse, a spontaneous model of SLE, exhibits higher leptin levels than control mice, and higher leptin levels are associated with higher disease activity. In NZBWF1 mice, leptin antagonist causes a delay in the onset of disease. Most clinical research has indicated that SLE patients have elevated leptin levels [19].

### 2.4 Role of Adiponectin

Several research teams discovered adiponectin, a 30‐kDa protein released by adipose tissue, in the mid‐1990s. Adiponectin's structural makeup is comparable to that of the complement component C1q. It consists of an N‐terminal signal sequence, a collagen‐like domain, a C‐terminal globular domain and a cysteine‐rich variable region that is required for multimer formation. Adiponectin forms a trimer through the development of a triple helix by the collagen domains and hydrophobic contacts between the globular domains. It then forms multimers composed of four to six trimers through oligomerization via interchain disulfide linkages [20].

### 2.5 Immune Function

Immune cells have AdipoR1, AdipoR2 and T‐cadherin on their surface. Depending on which receptor type is triggered on the cell, adiponectin may have different effects on immune function. Adiponectin stimulates the secretion of IL‐1 receptor antagonist and IL‐10, two anti‐inflammatory mediators, while suppressing the release of TNF‐α and IL‐6 in monocytes/macrophages. Mice lacking adiponectin display increased amounts of classically activated M1 macrophages in their adipose tissue, along with heightened quantities of TNF‐α, IL‐6 and MCP‐1 in these M1 macrophages [20]. Adiponectin's impact on T cells has been brought to light in a number of recent investigations. The frequency of IFN‐γ^+^ and IL‐17^+^ T cells is enhanced when naïve CD4^+^ T cells are grown in the presence of adipocytes from mice given a high‐fat diet; however, this is not the case when the cells are cultured in the presence of adipocytes from mice fed a normal diet. Subsequent research revealed that in mice given a regular diet, the decrease in T cells generating IL‐17 and IFN‐γ was caused by adipocyte‐derived adiponectin [20].

### 2.6 Adiponectin and Autoimmunity

Adiponectin is increased in several autoimmune illnesses, including SLE, in contrast to obese conditions, where circulating levels are lower than those of healthy controls. It is possible that the chronic elevations in inflammatory cytokines associated with some autoimmune disorders are what cause the increase in adiponectin in these conditions. Adiponectin‐deficient mice were created in the MRL/lpr model of SLE in order to investigate the function of adiponectin in SLE. Compared to MRL/lpr mice that had adiponectin, the animals displayed an exacerbation of the inflammatory phenotype, including lymphadenopathy, splenomegaly and elevated autoantibodies [21].

### 2.7 Overview of Resistin

Resistin, a protein produced by adipocytes and implicated in obesity‐related insulin resistance, was first discovered in obese mice. The 12.5 kDa protein resistin is abundant in cysteine residues and comprises a conserved C‐terminus, a variable domain and a signal peptide. A high‐molecular‐weight hexamer is generated by interchain disulfide connections, established by cysteine residues that enable its assembly into a trimer [22].

### 2.8 Resistin and Immune Function

In humans, resistin seems to have a more direct correlation with the immune system. In vitro, resistin's interaction with CAP1 on human monocytes induces the production of inflammatory cytokines via an NF‐kB‐dependent mechanism. Although the mechanism by which resistin functions via TLR4 remains incompletely elucidated, its interaction with TLR4 may also facilitate the release of proinflammatory signals. Proinflammatory cytokines, including CRP, TNF‐α, IL‐1β and IL‐6, can stimulate the release of resistin from infiltrating monocytes and peripheral blood mononuclear cells (PBMCs). Most investigations indicate that resistin exerts a proinflammatory effect, though certain studies have identified alternate functions for it. Resistin induces human dendritic cells (DCs) generated from monocytes to produce diminished cytokines and absorb reduced antigen, hence facilitating the proliferation of Tregs. At present, scant information exists on the role of adipokines in B lymphocytes, NK cells and neutrophils [21, 22]. Serum resistin has been correlated with proinflammatory cytokines, C‐reactive protein, total IgG and other inflammatory markers in systemic lupus erythematosus (SLE). Resistin has been examined concerning systemic lupus erythematosus (SLE), with a study correlating increased SLE disease activity scores and resistin levels to inflammatory markers in SLE [23].

### 2.9 Adipsin

The liver is the primary site of synthesis for other soluble complement components, whereas adipose tissue is the primary producer of adipsin. The role of complement in autoimmune disease has been well studied, and autoimmune disease is frequently caused by deficits in early complement components. Like adiponectin, adipsin is also overexpressed in a number of autoimmune disorders. Mice deficient in Factor D or adipsin exhibit reduced activity of SLE disease and less renal damage in the MRL/lpr model of SLE. According to reports, patients with SLE who also have renal symptoms have higher levels of adipsin than healthy controls do [21, 22, 23].

### 2.10 Chemerin

Chemerin is found in plasmacytoid DCs within SLE skin lesions, in the endothelium of dermal blood arteries and in the lesions themselves. Furthermore, the kidneys of SLE patients with lupus nephritis produce chemerin locally, which attracts CMKLR1^+^ plasmacytoid DCs to the kidneys. Nevertheless, when circulating chemerin levels were evaluated in SLE patients, a clinical investigation found no difference between the patients and the healthy control group [24, 25].

## 3 Gut Microbiota Dysbiosis: Linking Obesity and Lupus

**FIGURE 2:**
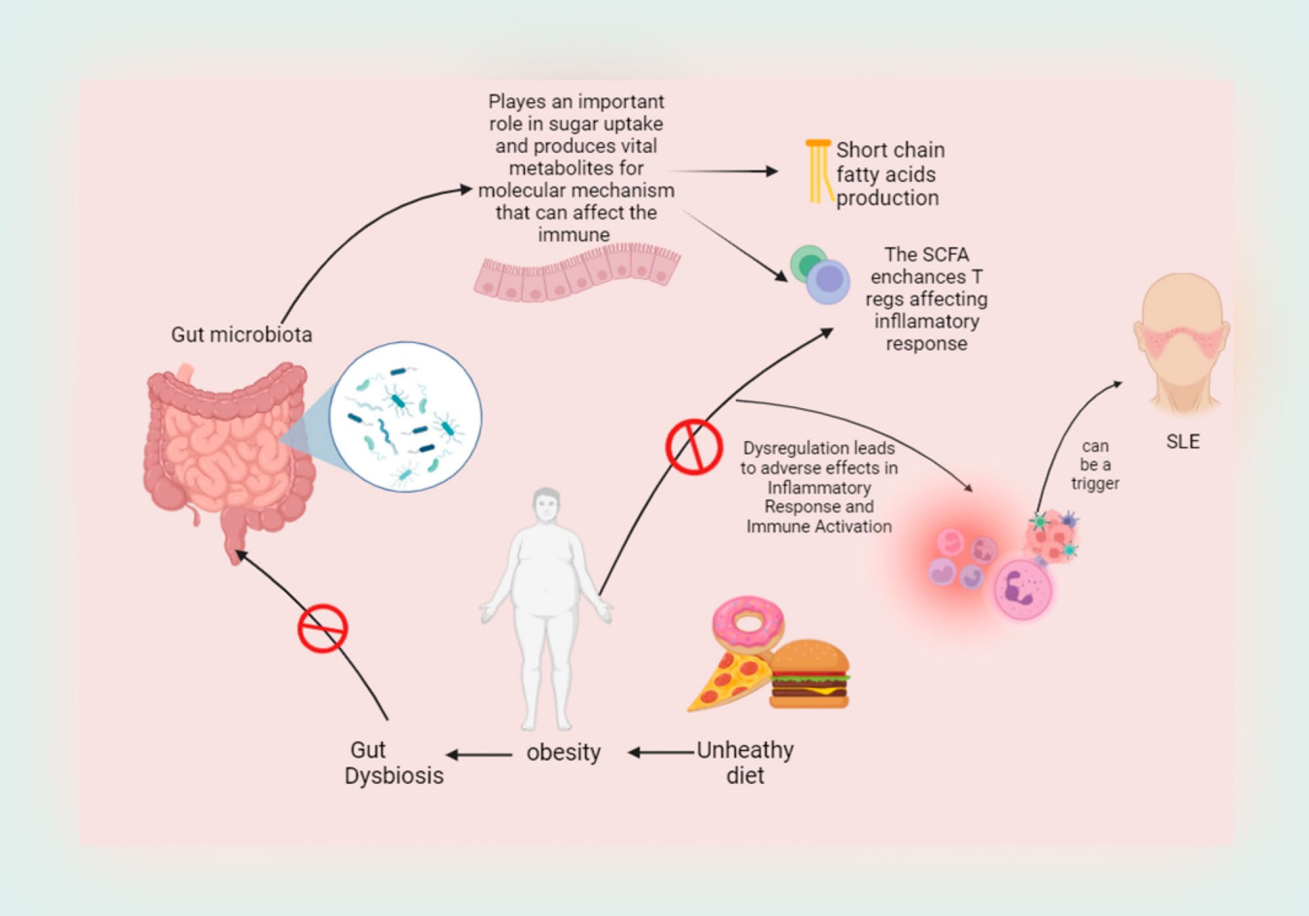
This figure demonstrates how obesity‐induced gut microbiota dysbiosis facilitates the advancement of systemic lupus erythematosus (SLE) by exacerbating inflammation, impairing immunological function and intensifying autoimmunity, consequently associating obesity with more severe lupus manifestations. Obesity‐induced gut dysbiosis promotes SLE progression by enhancing inflammation, immune dysfunction, and autoimmunity.

As shown in Figure 2, systemic lupus erythematosus (SLE), obesity and dysbiosis of the gut microbiota are closely related, as recent studies have shown. An imbalance in the gut microbial populations, or gut microbiota dysbiosis, is a major contributing factor to autoimmune illnesses such as SLE. Increased gut permeability results from altered gut microbial composition, which is commonly the result of high‐fat diets seen in obese patients. According to Candelli et al. [26], this permeability makes it possible for dangerous compounds like lipopolysaccharides (LPS) to enter the bloodstream and cause systemic inflammation, which is a key factor in the development of insulin resistance and type 2 diabetes [27]. Research shows people with SLE who have gut dysbiosis produce more pro‐inflammatory cytokines and have a compromised immune system. This imbalance accelerates the course of SLE by exacerbating autoimmunity. One potential treatment approach for SLE symptoms is to target the gut flora in order to alleviate the symptoms (Patra et al.) [28].

Additionally, the generation of short‐chain fatty acids (SCFAs), which are essential for energy balance and immunological modulation, is disrupted by dysbiotic gut flora in obese patients. Inflammation and metabolic issues are a result of this disruption. Breton et al. [29] highlight the potential of next‐generation probiotics to restore balance of the gut microbiota and lessen the negative effects of obesity [30]. Collectively, these results highlight the intricate relationship that exists between obesity, dysbiosis of the gut microbiota and autoimmune disorders such as SLE. This suggests that treating the gut microbiota may have positive therapeutic effects [27].

## 4 Epigenetic Modifications: Bridging the Gap Between Obesity and Lupus Susceptibility

Recent developments in the field of epigenetics shed light on the potential link between obesity and an increased risk of autoimmune illnesses like lupus. These can be considered a major public health concern and in‐depth research for these is an absolute necessity. Epigenetics deals with and describes the modifications in gene expression that occur without changing the DNA sequence, mainly as a result of non‐coding RNAs, histone modification and DNA methylation. Environmental factors, such as nutrition and obesity, can impact these adjustments, resulting in notable changes to immune function and inflammatory responses. Epigenetic alterations generated by obesity, including aberrant DNA methylation and altered histone acetylation, can result in the dysregulation of immunological tolerance and stimulate the activation of autoreactive immune cells, hence aggravating the course of systemic lupus erythematosus (SLE). These epigenetic modifications may perpetuate chronic inflammation and autoantibody synthesis, underscoring a vital connection between metabolic conditions and lupus vulnerability [29].

### 4.1 Effect of Epigenetic Modifications in Lupus

T cell deoxymethylcytosine concentration is 17% lower in patients with active SLE, and in vivo SLE‐like illness and autoreactivity are caused by inhibiting T cell DNA methylation [31]. It has been demonstrated that animals receiving CD4^+^ T cells treated with demethylating drugs, such as procainamide and hydralazine, have an illness resembling SLE in mouse models of drug‐induced lupus erythematosus [32]. These medications also cause autoreactivity in T cell lines that have been cloned and prevent T cell DNA methylation. Without the need for an external antigen, CD4^+^ T lymphocytes treated with the DNA methylation inhibitor 5‐azacytidine develop autoreactivity and react to self‐class II MHC. Defective DNA methylation and CD70 upregulation in CD4^+^ T cells were seen in lupus‐prone MRL/lpr mice [33].

All leukocytes express lymphocyte function‐associated antigen‐1 (LFA1), a heterodimer made up of the beta 2 chain (ITGB2) and integrin alpha L (ITGAL). Sequences surrounding the ITGAL gene promoter region in T cells from SLE patients were shown to be demethylated, indicating a potential mechanism for LFA‐1 overexpression on an autoreactive subset of T cells. T cell autoreactivity in SLE is believed to be linked to the overexpression of LFA‐1 and CD70 (TNFSF7), which in turn facilitates autoantibody release by B cells [34]. Patients with SLE may have persistently hypomethylated interferon genes and interferon‐regulated genes in their CD4^+^ T cells, CD19^+^ B cells, CD14^+^ monocytes and neutrophils. This mechanism is linked to abnormalities in the signalling of the extracellular signal‐regulated kinases (ERK) pathway, which in turn results in the downregulation of the methyltransferase DNMT1. According to these investigations, hypomethylated T cells produced by ERK pathway or DNA methyltransferase inhibitor therapy are enough to cause an illness resembling SLE [35].

In a transgenic mouse model, it was recently demonstrated that female mice with an inducible ERK deficiency acquired SLE‐like symptoms, but not male mice, indicating an ERK‐dependent female susceptibility for SLE [36]. Gorelik et al. linked decreased protein kinase C delta (PKCδ) phosphorylation to the SLE ERK pathway dysfunction. Genes linked to inflammation (CD40LG), the cytokine pathway (IL‐4, IL‐6, IL‐10, IL‐13 and IL1R2, respectively), and cell lysis (perforin) have also been found to be demethylation targets in SLE. These genes can all exacerbate inflammation by boosting the immune system [37]. The increase of the transcription regulatory factor cAMP‐responsive element modulator alpha (CREMα) in the T cells of MRL/lpr mice that are prone to lupus and patients with SLE is another example [38]. By binding to the CRE site in the gene promoter region, it attracts DNA methyltransferase DNMT3A, which aids in epigenetic remodelling. Afterwards, CpG hypomethylation is mediated by DNMT3A, which also modifies the CD8 cluster and silences IL2 and IL17A [39]. Conversely, recent research has demonstrated that an elevated histone H3 lysine 27 trimethylation enrichment at the promoter of haematopoietic progenitor kinase 1 (HPK1) in SLE CD4^+^ T cells (in comparison to controls) suppresses HPK1 expression and has a role in promoting autoimmunity in SLE [40]. But the consequences of DNA methylation are not limited to the molecule itself. DNA methylation acts either cooperatively or antagonistically on the various modifications of histone proteins because it keeps chromatin in a compacted and hence more inactive shape. For example, the chromatin of the hypomethylated CpG island is poor in linker histones and enriched in hyperacetylated histones [41]. Additionally, DNA methylation may shield people against autoimmune illnesses like SLE [42]. As women age, their chances of developing SLE or other sex‐related autoimmune disorders may decrease due to the hypermethylation of the oestrogen receptor, with each new gene discovered that is unregulated due to DNA de‐methylation [43].

### 4.2 Epigenetics and Obesity

Recent evidence associates obesity with the advancement of systemic lupus erythematosus (SLE) via metabolic alterations that enhance inflammation, immunological dysregulation and gut dysbiosis. Obesity is linked to epigenetic modifications, including CpG methylation and miRNA dysregulation during adipocyte development, which may influence immunological responses. It is proposed that epigenetic reprogramming induced by obesity is a crucial factor in the aetiology of systemic lupus erythematosus (SLE). Consequently, research ought to explore metabolic and epigenetic therapies to rectify obesity‐associated immune dysregulation in systemic lupus erythematosus (SLE) [44]. These changes impact their ability to metabolise fat and insulin sensitivity in adipose tissue, ultimately leading to β‐cell malfunction [44]. These epigenetic changes, known as metabolic memory, cause chronic inflammation even in the absence of the causative stressors (high‐fat diet and sedentary lifestyle). This ultimately results in arterial damage and the emergence of cardiovascular disease (CVD), which impairs patients' immune systems and increases their risk of infections, morbidity and death [45]. Blood cells and adipose tissue from obese individuals have been shown to exhibit variations in DNA methylation at the HIF3A gene [46]. In subjects with obesity of all ages, reprogramming of DNA methylation in PPARGC1A (the gene encoding PGC1, a master regulator of biogenesis and mitochondrial function) as well as hypermethylation of the proopiomelanocortin (POMC) promoter of intron 2/exon 3 in the melanocortin system have also been observed. These findings suggest that mitochondrial function plays a vital role in the development of obesity [47].

Histone alterations are another epigenetic mechanism connected to obesity, as previously mentioned. H3K4 is one of the histones associated with TNF‐α and IL6, which are proinflammatory cytokines that affect insulin signalling. Adipogenesis, which includes mechanisms like insulin regulation and hyperglycaemia, is regulated by miRNAs. About 20 miRNAs affect the release of CCL2, a gene associated with proinflammatory cytokines like TNF‐α and IL‐1 β. This gene's low regulation results in adipose inflammation, which raises the release of proinflammatory cytokines including TNF‐α and IL1β, which are involved in the pathogenesis of type 2 diabetes [48].

## 5 Metabolic Reprogramming and Autoimmunity: Insights From Obesity‐Associated Lupus Models

**FIGURE 3:**
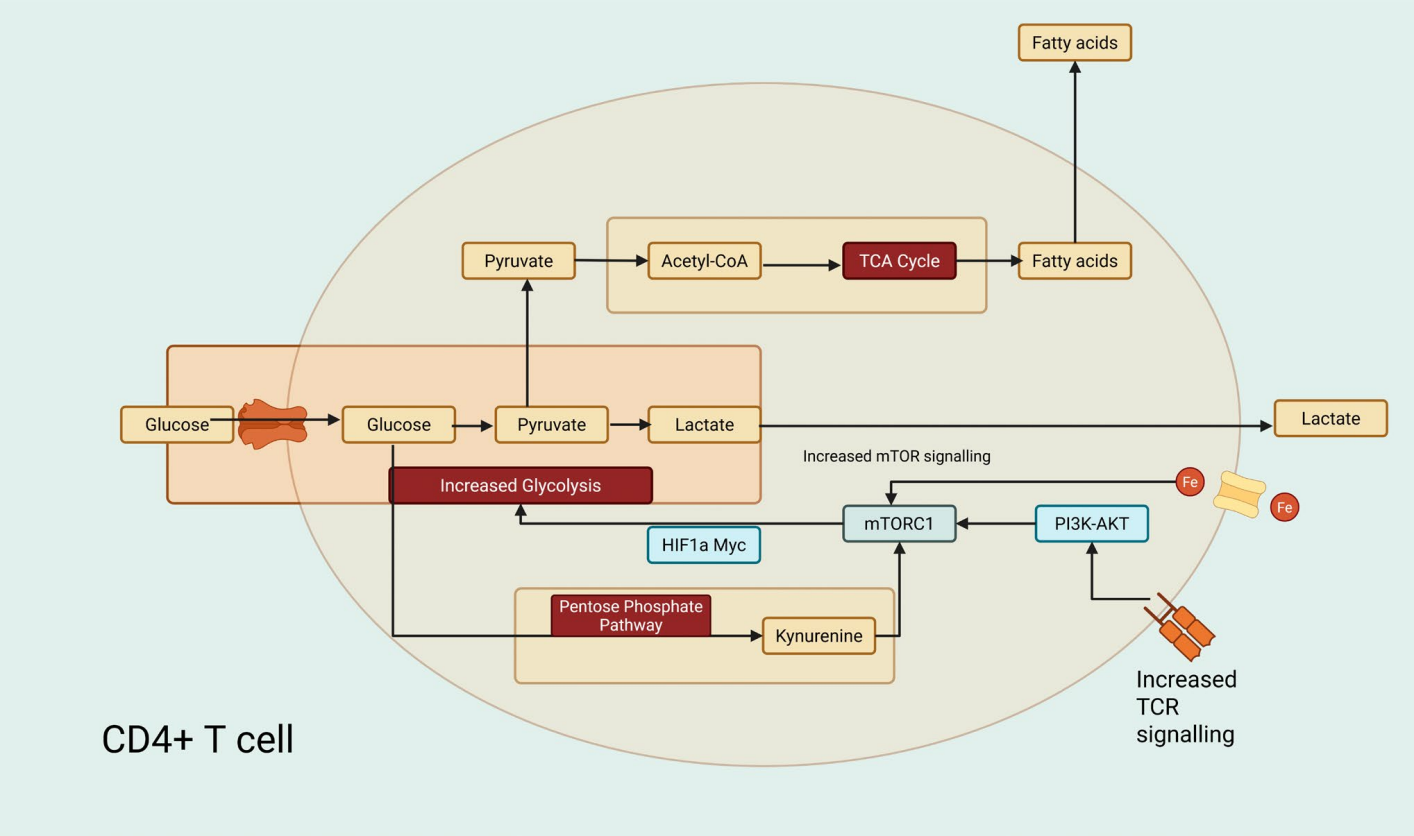
Obesity‐driven metabolic reprogramming impairs immune cells, triggering autoimmunity and inflammation that worsen disease.

Enhanced glycolysis, or the conversion of glucose to pyruvate, is a characteristic shared by CD4^+^ T cells from lupus‐prone animals and individuals with SLE. Pyruvate is either further converted into lactate, which is released by the cell, or it enters the TCA cycle to produce NADH and FADH2. In order to produce ATP through OXPHOS, NADH and FADH2 will subsequently join the electron transport chain (ETC). The PPP can also be used to metabolise glucose, which results in kynurenine buildup and the activation of mTORC1, the mechanistic target of rapamycin complex 1. Moreover, TCR activation can trigger mTORC1 via the PI3K–AKT pathway. By means of HIF‐1α and the proto‐oncogene protein Myc, mTORC1 activation through TCR activation can cause glucose metabolism, which further exacerbates mitochondrial dysfunction. CD4^+^ T cells use fatty acid oxidation to use fats as a source of fats. Moreover, through enhanced endosomal recycling, activated T cells enhance iron absorption and the transferrin receptor (CD71), which supports cell activation and differentiation (Figure 3) [49].

With well‐established changes in signalling, cytokine generation, proliferation and other regulatory functions, T cells play a major role in the initiation and maintenance of autoimmunity in SLE [50]. The T‐cell receptor (TCR) signalling of CD4^+^ T cells in SLE is rewired, resulting in an altered signalling phenotype. When the homologous Fcγ receptor chain replaces the reduced expression of the CD3ζ chain, downstream signalling occurs through Syk kinase rather than the typical CD3ζ partner Zap70 [51]. The lysosomal degradation of the CD3ζ chain occurs in lupus T cells due to elevated oxidative stress [52]. In both healthy donors and patients with autoimmune rheumatic illnesses, naïve T cells must signal via mTORC1 in order to polarise into type 1 T helper (TH1) and type 17 T helper (TH17) cells. Although it has been reported that mTORC1 inhibition encourages the growth of regulatory T cells (Treg), other evidence points to mTORC1 as a necessary component of Treg suppressive activity [53].

TFH cell differentiation and lupus‐like illness are induced in animal models by inhibition of AMP‐activated protein kinase (AMPK) and subsequent activation of mTORC1, even though CD4+ follicular helper T (TFH) cells do not activate the mTORC1 pathway during viral infection [54]. Patients with proliferative lupus nephritis have been observed to have an expansion of CXCR5^−^CXCR3^+^PD1hiCD4^+^ helper T cells in their peripheral blood and renal tubulointerstitial regions. These cells are distinct from TFH cells. Due to reverse electron transport fueled by succinate, it was discovered that these cells accumulated mitochondrial ROS, actively stimulating B‐cell activation through the supply of succinate and IL‐10 [55]. Numerous factors, including mitochondrial failure, activation of the pentose phosphate pathway (PPP), elevated transaldolase activity, and accumulation of kynurenine, a tryptophan metabolite with immunological modulatory properties, have been linked to mTORC1 activation in SLE T cells [56]. T‐cell function has also been demonstrated to be directly influenced by iron metabolism. Through enhanced endosomal recycling, activated T cells raised the transferrin receptor (CD71) and iron absorption, two characteristics that were amplified in lupus T cells [57]. Transferrin receptor blockade resulted in lower intracellular iron levels and mTORC1 signalling, which in turn suppressed TH1 and TH17 cells but promoted Treg development and was linked to a less severe course of the disease in lupus‐prone mice [58].

The generation of cytokines and effector activities by T cells is significantly influenced by glycolysis. A characteristic of CD4^+^ T cells from SLE patients and lupus‐prone mice is increased glycolysis [59]. Increased glucose absorption and glycolysis are correlated with GLUT1 expression, which is induced by T‐cell activation through TCR and CD28 stimulation. While the overexpression of GLUT1 in mice was linked to the generation of autoantibodies and cell activation, T cells in patients with SLE did not always exhibit this characteristic [60]. In a study examining the relationship between GLUT1 expression and SLE disease activity, the gene expression of GLUT1 did not significantly differ between healthy controls and SLE patients with low and high disease activity. However, compared to healthy controls or SLE patients with low disease activity (SLEDAI < 8), SLE patients with high disease activity (SLEDAI ≥ 8) had increased surface expression of GLUT1 on effector memory CD4^+^ T cells as shown by flow cytometry [61]. It has been demonstrated that in lupus‐prone animals, the hypoxic environment linked to renal tissue damage causes CD4^+^ and CD8^+^ T lymphocytes to overexpress hypoxia‐inducible factor‐1 (HIF‐1), which leads to metabolic reprogramming, enhanced effector function and resistance to apoptosis. Figure 1 provides an overview of CD4^+^ T‐cell metabolism and how it is impacted by SLE [62]. In CD8^+^ T lymphocytes, cholesterol plays a key role in controlling TCR signalling and effector activities. Through increased T‐cell receptor clustering and signalling in the immunological synapse, inhibiting cholesterol esterification in T cells by genetic ablation or pharmacological inhibition of acetyl‐CoA acetyltransferase 1, a crucial cholesterol esterification enzyme, led to increased T‐cell proliferation and activation of CD8^+^ but not CD4^+^ T cells. It is still unknown how lipid metabolism affects CD8^+^ T cells in SLE patients [63].

In 136 SLE patients, a large‐scale bulk RNA‐sequencing investigation of 27 immune cell types revealed unique transcriptome patterns linked to clinical characteristics like organ involvement and treatment responsiveness. It is interesting to note that distinct enrichment patterns between the fingerprints of cellular and metabolism‐related pathways were observed [64]. In memory CD8‐lineage cells, for example, TCA cycle genes were enriched in activity signatures. Conversely, disease‐activity signatures of TH1 cells and memory CD8‐lineage cells, as well as non‐T‐cell subsets such as NK cells, were enriched in ribosome and cell cycle pathways. The cell‐type‐specific analysis successfully identified the cell type from which these pathways originated and emphasised the role that immunometabolism plays in the early stages of illness development and aggravation [65].

## 6 Therapeutic Strategies Targeting Obesity‐Driven Pathways in Systemic Lupus Erythematosus

### 6.1 Targeting Metabolic Pathways

#### 6.1.1 mTOR Inhibitors

The initial strain of Rapamycin (RAPA) was identified as a water‐absorbing Streptomyces [66]. It is an allosteric mTOR inhibitor and a member of the macrocyclic lactone antibiotic class. It is an mTOR allosteric inhibitor that can create a FRB domain with the FKBP12 protein to block mTOR activity, stop the cell cycle at the G1 phase and trigger autophagy and apoptosis in cells [67]. Since 1999, RAPA has been utilised as a successful treatment to stop acute organ transplant rejection and to slow the development of chronic allograft lesions. RAPA may reduce the synthesis of autoantibodies, enhance proteinuria and increase the longevity of lupus‐prone animals, according to data [68]. It has likewise been used for exploring the beneficial effects of autoimmune diseases. Increasing clinical results proved RAPA facilitates disease alleviation, organ protection and extends life span [69, 70]. The safety and effectiveness of RAPA in treating SLE were validated by a recent study published in the Lancet. The study was a single‐arm, open‐label, phase 1/2 trial [71].

According to its findings, RAPA was used for a 12‐month period to treat 29 patients with active SLE who were either intolerant or resistant to conventional medications. During this time, there were a few side effects to be aware of, including severe oral ulcers, a moderate decline in neutrophil counts and temporary hyperlipidemia. In 16 individuals, the British Isles Lupus Assessment Group disease activity index and systemic lupus erythematosus disease activity index (SLEDAI) scores both markedly declined. In the meantime, it was discovered that RAPA increased the quantity of CD4^+^CD25^+^Foxp3^+^T cells and CD8^+^ memory T cells in the peripheral blood, preventing CD4^+^ and DN T cells from producing IL‐4 and IL‐17, which helped active SLE patients experience a reduction in their condition [72]. As there are not many publications on this topic, it is currently considered that RAPA activates mTORC2, which is secondary. Overall, the various effects that rapamycin may have upon mTOR inhibition in the pathophysiology of lupus are responsible for either the drug's negative effects or its therapeutic success [70].

#### 6.1.2 AMPK Activators Metformin

A study done by Fangfang Sun et al. showed that metformin can be used for the treatment of SLE [73]. Well‐known for its hypoglycaemic properties, metformin has been shown to have both AMP‐activated protein kinase (AMPK)‐dependent and AMPK‐independent anticancer, antiaging, cardioprotective, anti‐inflammatory and immunomodulatory effects. Proinflammatory cytokine release, oxidative stress and immune cell activation and proliferation have all been demonstrated to be reduced by metformin [74]. Metformin administration reduced kidney damage, improved kidney function and improved mice survival in experimental lung necrosis (LN) models by suppressing the systemic and intrarenal inflammatory response in the kidney, primarily through necroptosis and inflammasome activation through AMPK‐mediated inhibition of STAT3. These findings are consistent with a recent in vivo study testing metformin as a treatment for SLE [75].

#### 6.1.3 Fatty Acid Oxidation Modulators

A class of drugs known as PPAR agonists targets the peroxisome proliferator‐activated receptor. In addition to its clinically valuable ability to decrease cholesterol, gemfibrozil, an agonist of PPAR‐α, has been shown to prevent the release of cytokines that promote inflammation [76]. During deadly influenza infections, a considerable number of TNF‐producing dendritic cells (tipDC) develop in the lungs. It is here that they obtain, process and present influenza antigen to CD8 T lymphocytes in conjunction with MHC class I antigen [77]. A study done by Hasni et al. showed that, when PGZ was used in place of a placebo, there was a noteworthy decrease in the Cardio‐Ankle Vascular Index, which gauges arterial stiffness. With PGZ, a number of metabolic markers improved, including lipoprotein profiles and insulin resistance. In addition, PGZ dramatically reduced the levels of circulating neutrophil extracellular traps as compared to a placebo. The majority of side effects were modest and went away when the dosage of PGZ was lowered [78]. In patients with SLE, PGZ significantly improved cardiometabolic indices and vascular stiffness while also being well tolerated. The findings imply that PGZ should be investigated more as a modulator of the risk of cardiovascular disease in individuals with SLE [78].

#### 6.1.4 Anti‐Inflammatory Therapies

It has been proven that leptin promotes SLE. It can be theorised that leptin modulators may help in treating SLE. Since there are limited research studies done on this topic, there is not much information available on this topic. By raising adiponectin levels, these medications can lessen systemic inflammation and increase insulin sensitivity. Given that adiponectin levels are frequently dysregulated in individuals who suffer from obesity with SLE, this may be especially helpful [79]. A developing issue with infliximab therapy is the emergence of autoimmunity. According to reports, infliximab treatment is linked to the induction of histone in 21% of patients, ds DNA in 32.5% of patients, and ANA in 56.8% of patients. Even while autoimmunity is highly prevalent, clinically significant SLE is incredibly uncommon. Clinical SLE was linked to the development of ANA, ds DNA IgM, histones and female sex in the six instances that were reported [80]. A 1990 investigation using NZB/W F1 mice, an animal model of systemic lupus erythematosus (SLE), revealed a potential involvement for IL‐6 in the immune complex‐mediated glomerulonephritis pathogenesis [81]. Additionally, patients with SLE or lupus nephritis have higher serum and urine IL‐6 concentrations, which are correlated with disease activity. Treatment with tocilizumab improved disease activity in an open‐label phase I research with 16 SLE patients; notably, arthritis improved in all seven patients who had it at baseline and disappeared in four of them [82]. Even after accounting for the drop in total IgG titres after tocilizumab treatment, levels of antibodies to double‐stranded DNA decreased112. These modifications, along with a drop in the quantity of plasma cells in circulation, indicated that IL‐6 suppression had a particular effect on B cells that produce autoantibodies. Nevertheless, additional research using sirukumab failed to show that blocking the IL‐6 pathway in patients with SLE or lupus nephritis had a clinically significant advantage [83]. Further clinical advancement in SLE has been restrained by these contradictory results. Further research is necessary to determine whether IL‐6 suppression is beneficial for some SLE symptoms but not others [84].

#### 6.1.5 Epigenetic Therapies

Numerous genes implicated with SLE have been demonstrated to have their imbalanced expression corrected by Histone Deacetylase Inhibitors (HDACi). It was noted that Foxp3 gene expression, Treg cell generation and their suppressive capacity were all elevated by HDAC inhibition in vivo. The impact of HDAC9 on Treg cell function is very fascinating. Research has indicated that HDAC9 has demonstrated a significant role in controlling Foxp3‐dependent repression [85]. The pathophysiological implications, molecular mechanisms and possible therapeutic targets at different sites are displayed in Table 1.

**TABLE 1:** Molecular mechanisms, pathophysiological implications and potential therapeutic targets across different sites.

Sl. No.	Targeted site	Key molecular mechanisms	Pathophysiological implications	Potential therapeutic targets	References
	Obesity‐driven dysregulation of immune1omeostasis	Immune cells that have undergone metabolic reprogramming begin to produce energy through glycolysis rather than fatty acid oxidation. Immune responses are impacted by fatty acid oxidation inhibition	Obesity‐related immune cell activation over time may worsen autoimmune diseases like lupus by causing more inflammation and tissue damage	Targeting immune cell metabolic pathways, such as fatty acid oxidation or glycolysis inhibitors, is one avenue for potential treatment	[1, 6]
2.	Adipose tissue inflammation: implications for lupus pathogenesis	Systemic inflammation may result from inflamed adipose tissue producing more pro‐inflammatory cytokines and adipokines	Adipose tissue can produce inflammatory signals that worsen systemic inflammation in SLE patients, accelerating the course of the illness and its flare‐ups	Treatment strategies could focus on reducing inflammation in adipose tissue or pro‐inflammatory cytokine signalling	[1, 5]
3.	Adipokines and cytokine crosstalk in lupus development	The immune response can be boosted by dysregulated adipokine and cytokine signalling, which can result in a rise in autoantibody synthesis and systemic inflammation	Tissue and organ damage in SLE is a result of immunological dysregulation brought on by aberrant cytokine and adipokine signalling	In SLE, focusing on cytokine and adipokine pathways may help lower inflammation and stop the disease's progression	[1, 4]
4.	The role of leptin signalling in lupus pathophysiology	Leptin contributes to the autoimmune response in SLE by influencing immune cell activation and promoting T‐cell proliferation	By encouraging immune cell activation and maintaining autoimmunity, elevated leptin levels in obesity can exacerbate lupus	In lupus patients, leptin signalling modulation may be helpful in controlling autoimmune reactions and lessening the severity of the condition	[1, 3]
5.	Gut microbiota dysbiosis: linking obesity and lupus	Increased gut permeability brought on by altered gut microbiota permits LPS access into the circulation. T cell dysregulation and persistent inflammation are caused by increased intestinal permeability and the dissemination of bacterial products	The condition of gut dysbiosis accelerates the evolution of SLE by aggravating insulin resistance and autoimmune, as well as systemic inflammation	Probiotics or prebiotics can help restore the equilibrium of the gut flora, which can lower inflammation and autoimmune. Probiotics of the next generation to balance the gut microbiota, dietary changes to improve the secretion of SCFAs, and targeting particular microbial populations	[1, 3, 27, 28, 30]
6.	Epigenetic MODIFICATIONS: BRIDGING OBESITY AND LUPUS SUSCEPTIBILITY	Immune cell activity may be altered by epigenetic modifications brought on by long‐term inflammation and metabolic stress from obesity	Epigenetic changes have the potential to cause immunological dysregulation, which raises the risk of developing lupus	In obese lupus patients, targeting epigenetic regulators like HDAC inhibitors may help control immunological responses	[6, 7]
7.	Metabolic reprogramming and autoimmunity	Dysregulation of TLR7 and TLR8 via inflammation exacerbates inflammatory responses and advances metabolic syndrome	Increased TLR7 signalling in obese people with metabolic syndrome may hasten the start of SLE and impede its advancement	Based on evidence from animal models, inhibiting TLR7/8 signalling may lower the incidence of metabolic syndrome and lupus	[9, 10]
8.	Therapeutic strategies targeting obesity‐driven pathways	Three major mechanisms induced by obesity in SLE include enhanced TLR signalling, dysregulated adipokines, and chronic inflammation	In obese patients, metabolic problems and lupus symptoms develop due to immunological dysregulation and chronic inflammation	TLR7/8 inhibitors, inflammatory reducers, or medications that target adipokine signalling are examples of possible treatments	[10,11]
9.	Obesity and immune cell plasticity	The flexibility of immune cells is altered by obesity, which results in immune cell malfunction and an aggravation of autoimmunity	Obesity‐related changes in immune cell plasticity may worsen SLE symptoms and prolong inflammatory responses	Therapeutic approaches to reestablish normal immunological function can focus on immune cell metabolism and plasticity	[6, 7]
10.	Inflammatory mediators in obesity and lupus	Systemic inflammation is caused by dysregulation of adipokines (adiponectin, leptin), as well as cytokine release in adipose tissue	Adipokines and inflammatory cytokines exacerbate autoimmunity in SLE patients, facilitating disease development and heightened severity	Reducing inflammatory and autoimmune responses may be achieved by modifying cytokine release or focusing on adipokine signalling, particularly leptin	[12, 14, 15]
11.	Role of insulin resistance in lupus	Impaired insulin signalling as a result of dysregulation of IRS‐1 and IRS‐2; elevated synthesis of pro‐inflammatory cytokines (TNF‐α, IL‐6) as a result of insulin resistance	Insulin resistance aggravates the disease and raises the risk of cardiovascular events and organ damage. It also has a role in inflammation and autoimmune in lupus	TNF‐α inhibitors, AMPK activators, insulin sensitizers (like Metformin), and lifestyle modifications	[73, 80]
12.	Genetic predisposition to obesity and lupus	Susceptibility to obesity and lupus is influenced by SNPs in obesity‐related genes (e.g., FTO, MC4R) and epigenetic alterations (e.g., DNA methylation, histone modifications)	Genetic predisposition raises the risk of obesity, and because obesity causes inflammation, this may then increase the risk of developing lupus	Medicines that target certain genes, changes to lifestyle, and epigenetic therapies (such HDAC inhibitors)	[85, 86]
13.	Role of oxidative stress in obesity‐associated lupus	Reactive oxygen species (ROS) generation is increased as a result of poor antioxidant defences and metabolic dysfunction; oxidative stress‐induced activation of the NF‐κB and MAPK pathways	In lupus, oxidative stress plays a role in tissue damage and inflammation, which feeds the immunological dysregulation cycle and exacerbates symptoms of the illness	MAPK inhibitors, NF‐κB inhibitors, and antioxidants such N‐acetylcysteine	[51, 53]
14.	Role of MicroRNAs in Obesity and Lupus	In immune cells and adipose tissue, dysregulated microRNAs (miR‐155, miR‐146a) modify the control of inflammation and metabolic processes in lupus	Changes in miRNA profiles may exacerbate autoimmune activity in lupus by influencing T cell activation, immunological responses, and adipokine release	Treatments that target particular miRNAs and lifestyle changes to bring back the equilibrium of miRNAs	[59, 82]
15.	Interplay between hormones and lupus	White adipose tissue secretes leptin, which influences T‐cell differentiation and cytokine levels in immune cells	Leptin contributes to immunological dysregulation in lupus by increasing Th1 and Th17 responses and decreasing Th2 and Treg responses	In lupus, inhibiting leptin receptors or adjusting leptin signalling may be helpful in controlling inflammation and autoimmune	[15, 16, 18]
16.	Impact of sleep disturbances on lupus and obesity	Interruptions to circadian cycles that impact inflammatory markers (e.g., TNF‐α, IL‐6), cortisol levels, and sleep architecture that elevate sympathetic nervous system activity.	Sleep problems in lupus can worsen disease activity and increase fatigue, mood swings, and cardiovascular risks by aggravating inflammation and autoimmune	Cognitive behavioural therapy, pharmaceutical medications for sleep problems, and sleep hygiene interventions	[87, 88]
17.	Inflammatory Pathways in Cardiovascular Risks in Lupus	Chronic inflammation causes endothelial dysfunction and increased platelet activation. It also activates inflammatory pathways such as IL‐1, IL‐6, and TNF‐α, as well as oxidative stress.	Patients with lupus who experience chronic inflammation have higher cardiovascular risks, which can result in myocardial infarction and other cardiovascular problems	Statins, anti‐inflammatory drugs, and lifestyle changes (diet, exercise, etc.)	[86, 89]
18.	Role of neuroinflammation in obesity‐associated lupus	Increased levels of pro‐inflammatory cytokines (IL‐1β, IL‐6), activation of astrocytes and microglia, and changes in neuropeptide signalling that affect mood and cognition	In lupus, neuroinflammation exacerbates mood disorders, pain perception, and cognitive dysfunction, making quality of life and disease management more difficult	Neuroprotective agents, anti‐inflammatory therapies, cognitive behavioural therapy	[74, 83]
19.	Longitudinal studies of obesity and lupus	Finding the metabolic signatures and biomarkers linked to obesity in lupus patients; monitoring changes in inflammatory markers and body composition over time	Insights into the dynamic association between obesity and lupus are provided by longitudinal studies, which can aid in determining the important times for intervention and risk assessment	Targeted treatments based on long‐term data and continuous metabolic health monitoring	[65, 81]
20.	Future Directions in Research on Obesity and Lupus	Investigation of the relationship between genes and environment, the function of the microbiota in obesity and lupus, and the creation of precision medicine strategies to target lupus's obesity‐related pathways	Improvements in research could result in more individualised treatment plans, a greater comprehension of the role fat plays in the pathophysiology of lupus, and better patient outcomes	Novel treatments that focus on pathways that have been uncovered, microbiome regulation, and precision medicine techniques	[64, 86]

#### 6.1.6 Lifestyle and Behavioural Interventions

The findings indicated that while exercise did not lessen disease activity, it could enhance HRQoL [87], reduce fatigue and lessen depressed symptoms in SLE patients [88]. The person who was evaluated also discovered that while exercise had no effect on disease activity or the prevention of organ damage, it could alleviate sensations of depression and exhaustion [90]. Non‐pharmacological methods are used in the therapy of lupus. Patients should wear protective clothing and use sunscreen with at least SPF 50 to prevent being in the sun.

In SLE, there is a higher prevalence of fibromyalgia and which is the propensity to react to sickness and psychosocial stress with weariness, a general rise in symptoms, and broad pain. Frequent stretching and exercise can help reduce fibromyalgia discomfort, exhaustion and cognitive problems. All‐cause mortality is increased by 2.4 times in lupus patients. Cardiovascular events are the leading cause of death in lupus, with infections and lupus‐related renal and respiratory problems coming in third and fourth [86]. There is a 2.66‐fold increase in the risk of cardiovascular events. To prevent early death, it is imperative to aggressively control modifiable cardiovascular risk factors, including those related to lupus (lupus activity, antiphospholipid antibodies, homocysteinemia, excessive corticosteroid use), as well as classic cardiovascular risk factors (smoking, obesity, diabetes mellitus, hypertension and dyslipidemia) [89]. Fifteen percent of patients had homocysteinemia, which is independently linked to cardiovascular risk, renal damage and fibrosis. Patients with antiphospholipid antibodies also have a greater rate of myocardial infarction and thrombosis. In lupus patients, hyperhomocysteinemia stands alone as a risk factor for cardiovascular disease [91]. Lupus patients frequently get infections, especially from encapsulated bacteria. The most frequent causative agents are typical organisms, but there is also a rise in opportunistic bacterial, mycobacterial, protozoal, fungal and viral infections. Pneumonia is prevalent in lupus patients and is linked to higher mortality, particularly when it is caused by *Streptococcus pneumoniae* . It is recommended to adhere to the pneumococcal and influenza vaccine schedule in addition to the local immunisation schedule [92]. The treatment section must be augmented to incorporate GLP‐1 analogues, which are emerging as efficacious drugs in the management of both obesity and diabetes mellitus. These medicines not only facilitate weight reduction and glycemic regulation but may also provide protective benefits against autoimmunity. The risk of fragility fractures and osteoporosis is increased with lupus. Notably, fracture risk calculators in lupus patients may be understated [93]. The goal of achieving optimal bone health should include quitting smoking, achieving optimal vitamin D levels, getting enough calcium from food rather than supplements, engaging in weight‐bearing exercise, avoiding corticosteroids, having bone density screenings based on risk, and receiving treatment with DHEA (never in men) and bisphosphonate when necessary [94, 95, 96, 97]. Future therapy techniques may enhance efficacy by personalising metabolic pathway‐targeted medicines, such as mTOR inhibitors and AMPK activators, according to patients' genetic and metabolic profiles to maximise outcomes and reduce unwanted effects.

Obesity is not present in all patients with SLE; however, it can exacerbate immunological dysfunction and inflammation. Our research identifies novel connections between metabolic alterations induced by obesity—such as modifications in the body's processing of glucose and lipids—and the onset of lupus. We emphasise that an imbalance in gut microbiota, particularly a reduction in beneficial bacteria and an increase in pathogenic ones, can exacerbate immunological complications in obese individuals with systemic lupus erythematosus (SLE). Our research reveals novel associations between metabolic alterations induced by obesity—such as modifications in glucose and lipid metabolism and the onset of lupus. We emphasise the increasing significance of gut microbiota dysbiosis, namely the reduction of microbial diversity and the proliferation of pro‐inflammatory species, in exacerbating immunological dysfunction in obese persons with systemic lupus erythematosus (SLE). These discoveries offer a novel viewpoint on the convergence of metabolic and microbial pathways that may exacerbate the severity of autoimmune diseases, hence creating opportunities for targeted therapies based on metabolism and the microbiome. Although the majority of individuals with systemic lupus erythematosus (SLE) are not obese, obesity is posited to play a role in the aetiology of SLE. This notion is substantiated by studies connecting obesity to chronic low‐grade inflammation, immune system dysregulation, and alterations in gut microbiota composition, variables that may collectively enhance disease activity and reduce remission durations. While this relationship is not universally applicable to all individuals, obesity‐related changes in adipose tissue and immune function are seen as potential factors in the evolution of SLE.

## 7 Conclusion

Recent research indicates that obesity aggravates systemic lupus erythematosus (SLE) by inducing metabolic dysregulation, chronic inflammation and immunological dysfunction, thus exacerbating disease progression. The immunological imbalance linked to obesity is exacerbated by dysbiosis of the gut microbiota and epigenetic modifications. We propose that obesity serves as a significant modulator of systemic lupus erythematosus (SLE) pathogenesis, and that addressing its underlying molecular mechanisms by pharmaceutical and lifestyle therapies may provide an innovative strategy for enhancing clinical outcomes in SLE. Therapies such as metformin and PPAR agonists demonstrate potential in mitigating inflammation and improving metabolic profiles, while anti‐inflammatory treatments and epigenetic approaches may provide additional avenues for management. Moreover, lifestyle modifications, including exercise and dietary changes, can enhance the quality of life for patients. Further research is essential to fully understand these interactions and optimise therapeutic strategies tailored to individual patient needs.

## Author Contributions

All authors participated in the conceptualization, literature review and composition of the text. R.K.P., V.R.I., V.B. and A.M.A. spearheaded the conceptual framework and orchestrated the manuscript preparation. R.K.P., A.B., H.K.D., S.K., M.K.S.J. and V.S. performed the literature review and contributed to the sections on metabolic reprogramming and gut microbiota. V.S. evaluated and revised the text for intellectual substance and scientific precision. All authors reviewed and endorsed the final manuscript.

## Conflicts of Interest

The authors declare no conflicts of interest.

## Data Availability

The data that support the findings of this study are available on request from the corresponding author. The data are not publicly available due to privacy or ethical restrictions.
